# Heparanase 2, mutated in urofacial syndrome, mediates peripheral neural development in Xenopus

**DOI:** 10.1093/hmg/ddu147

**Published:** 2014-04-01

**Authors:** Neil A. Roberts, Adrian S. Woolf, Helen M. Stuart, Raphaël Thuret, Edward A. McKenzie, William G. Newman, Emma N. Hilton

**Affiliations:** 1 Centre for Genomic Medicine and; 2 Centre for Paediatrics and Child Health, Institute of Human Development, Faculty of Medical and Human Sciences,; 3 Developmental Biology and; 4 Protein Expression Facility, Faculty of Life Sciences, University of Manchester, Manchester M13 9PT, UK

## Abstract

Urofacial syndrome (UFS; previously Ochoa syndrome) is an autosomal recessive disease characterized by incomplete bladder emptying during micturition. This is associated with a dyssynergia in which the urethral walls contract at the same time as the detrusor smooth muscle in the body of the bladder. UFS is also characterized by an abnormal facial expression upon smiling, and bilateral weakness in the distribution of the facial nerve has been reported. Biallelic mutations in *HPSE2* occur in UFS. This gene encodes heparanase 2, a protein which inhibits the activity of heparanase. Here, we demonstrate, for the first time, an *in vivo* developmental role for heparanase 2. We identified the Xenopus orthologue of heparanase 2 and showed that the protein is localized to the embryonic ventrolateral neural tube where motor neurons arise. Morpholino-induced loss of heparanase 2 caused embryonic skeletal muscle paralysis, and morphant motor neurons had aberrant morphology including less linear paths and less compactly-bundled axons than normal. Biochemical analyses demonstrated that loss of heparanase 2 led to upregulation of fibroblast growth factor 2/phosphorylated extracellular signal-related kinase signalling and to alterations in levels of transcripts encoding neural- and muscle-associated molecules. Thus, a key role of heparanase 2 is to buffer growth factor signalling in motor neuron development. These results shed light on the pathogenic mechanisms underpinning the clinical features of UFS and support the contention that congenital peripheral neuropathy is a key feature of this disorder.

## INTRODUCTION

Urofacial syndrome (UFS; previously Ochoa syndrome; OMIM #236730) is an autosomal recessive disease. It is characterized by incomplete bladder emptying during micturition. This is associated with a dyssynergia in which the urethral walls contract at the same time as the detrusor smooth muscle in the body of the bladder (1,2). Through post-natal life, individuals with UFS can suffer from urinary incontinence, urosepsis, damaged kidneys and, ultimately, renal failure. UFS is also characterized by an abnormal facial expression upon smiling, typically with downturned lateral aspects of the mouth (1–4). Individuals with UFS have nocturnal lagophthalmos, the lack of complete eyelid closure during sleep (5) and a more marked bilateral weakness in the distribution of the facial nerve has been reported (6). Newman (4) noted that 150 affected individuals with UFS had been reported in the medical literature available to 2013. The condition, however, may be underreported because the clinical severity is variable. For example, in families with more than one affected member, existing individuals have sometimes only been designated as having UFS after their more severely affected sibling was diagnosed. The congenital nature of certain aspects of the syndrome is evidenced by the fact that UFS newborns have a distorted expression when crying and, moreover, lower urinary tract dysmorphology (e.g. hydronephrosis and an enlarged bladder) can present before birth (7). Thus, at least some clinical features of UFS should be regarded manifestations of abnormal development.

In 2010, we and others showed that biallelic mutations in *heparanase 2* (*HPSE2*) cause UFS (7,8), with further cases subsequently reported (9,10). Most *HPSE2* mutations in UFS are predicted to be null mutations, with only one missense mutation reported to date (10). The severity of urinary tract and facial features varies among family members with the same mutation (4,7). *HPSE2* codes for heparanase 2, a protein with 44% identity and 59% similarity to the endoglycosidase heparanase (for clarity, henceforth referred to as heparanase 1), encoded by *HPSE1* (11). Heparanase 1 catalyses the cleavage of heparan sulphate (HS) sidechains from HS proteoglycans (HSPGs), cell surface and extracellular matrix macromolecules that bind to growth factors and structural matrix proteins, thereby regulating their activities (12,13). For example, heparanase 1 enhances fibroblast growth factor 2 (FGF2) availability (14) and signalling (15,16), the latter evidenced by increased amounts of phosphorylated extracellular signal-related kinase (pERK; 16). Mammalian heparanase 2 lacks endoglycosidase activity but it binds HS with a higher affinity than heparanase 1, inhibiting heparanase 1 activity by sequestering HSPG targets and preventing HS-mediated cellular uptake of heparanase 1, required for its enzymatic activation within lysosomes (17).

In 2013, we identified biallelic mutations in *leucine-rich repeats and immunoglobulin-like domains 2* (*LRIG2*) in UFS families lacking *HPSE2* mutations (18; OMIM #615112). As for most mutations in *HPSE2*, *LRIG2* mutations are predicted to be null (reviewed in 4). *LRIG2* encodes leucine-rich repeats and immunoglobulin-like domains 2 (LRIG2), one of a family of three mammalian LRIGs (19). LRIGs are transmembrane proteins which, based mostly from studies on LRIG1, are considered to modulate growth factor signalling by controlling receptor tyrosine kinase (RTK) turnover (20–22). Notably, clinical phenotypes are indistinguishable between *HPSE2*- and *LRIG2*-linked UFS so the encoded proteins are likely to have overlapping functions in development. It is plausible that, based on the limited published data regarding functions of heparanase 2 and LRIG2, clinical features of UFS arise from dysregulated actions of growth factors which bind RTKs and signal through ERK phosphorylation.

The exact cell types which are affected by *HPSE2* and *LRIG2* mutations in UFS are currently undefined, although a neurogenic basis for the syndrome has long been postulated (2). The UFS bladder resembles a dysfunctional ‘neurogenic’ organ which can result from spinal cord injury, yet UFS individuals lack overt spinal lesions (2; reviewed in 23). Ochoa (2) speculated that both the bladder and facial features of UFS were explained by one brainstem lesion affecting both the VII nerve nucleus supplying facial skeletal muscles and also the micturition centre that modulates bladder emptying. However, others (3) suggested that UFS bladder dysfunction as well as constipation, a variable feature of the syndrome, both result from defects in sacral spinal cord nuclei which send motor axons to skeletal muscle in the external bladder and anal sphincters. We recently showed that transcripts for both UFS genes are present in healthy mammalian bladders; moreover, HPSE2 and LRIG2 proteins were immunodetected in nerve fascicles growing into muscle layers of human foetal bladder walls (7,18). These latter observations provide evidence that peripheral nerves are involved in UFS.

In the current study, we generated a Xenopus model to begin to define the biological roles of heparanase 2 in vertebrate development. The data show that, in this species, this UFS molecule is required for the functional development of motor nerves emerging from the truncal neural tube. These results support the hypothesis that peripheral neuropathy is a key element of the human UFS clinical phenotype. Furthermore, we present evidence that depletion of heparanase 2 in frog embryos is associated with upregulation of *fgf2* and pERK, consistent with the hypothesis that *HPSE2* encodes a molecule which regulates actions of growth factors during normal development.

## RESULTS

### *Xenopus tropicalis* heparanase 2

Using the tblastn tool and the human heparanase 2 amino acid sequence, we searched the *Xenopus tropicalis* genome project database (JGI) for a putative heparanase 2 orthologue. We identified a 569 amino acid fragment match with homology from amino acid 24 of the human protein to the stop codon. From the corresponding Xenopus genomic sequence, we identified an in-frame ATG start codon starting 33 bp upstream and positioned 51 bp downstream of an in-frame stop codon; we annotated this ATG as the start codon for Xenopus heparanase 2, and this prediction concurs with that of Gnomon, the NCBI gene prediction tool (XM_002937183.2).

Synteny analysis of the region of the Xenopus genome encoding the putative heparanase 2 orthologue (Metazome; data not shown) revealed a syntenic block encompassing at least 15 genes (centred at *SLC25A28*/*GOT1*/***HPSE2***/*HPS1*/*LOXL4*) conserved between Xenopus, human and mouse genome sequences. Direct comparison of the human and Xenopus genomic sequences revealed perfect conservation of exon/intron boundaries (data not shown). This analysis confirms the identification of a Xenopus orthologue for heparanase 2.

**Figure 1.**
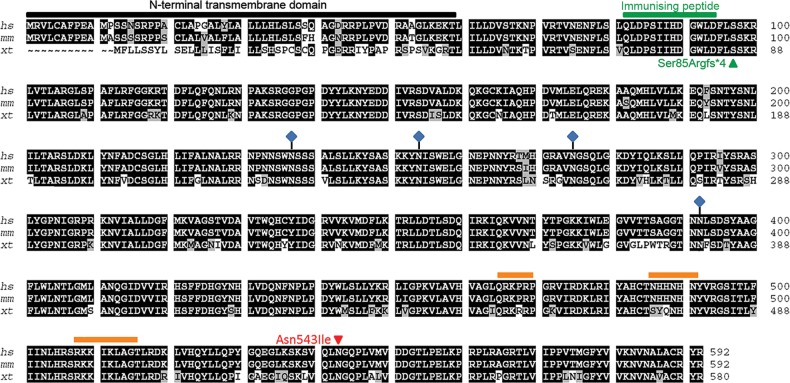
Alignment of the Xenopus heparanase 2 protein sequence with human and mouse orthologues. Xenopus heparanase 2 shares 80% identity (black highlight) and 88% similarity (black plus grey highlight) with human heparanase 2. The majority of non-conserved amino acids are in the N-terminal region (black bar), predicted to constitute a signal peptide. Putative HS-binding motifs are indicated by orange bars. Glycosylated amino acids are indicated by blue diamonds. Asn543, which undergoes a missense mutation in UFS (10), is conserved between species. The immunizing peptide used to generate the heparanase 2 antibody used in this study is indicated in green as is the nonsense mutation created by injection of the splice acceptor morpholino (MO1).

At the protein level, Xenopus heparanase 2 has 580 amino acids and shares 80% identity and 88% similarity with human heparanase 2 (Fig. 1), consistent with the proteins having conserved evolutionary function. The first 80 amino acids in the human N-terminal region show low conservation with the Xenopus sequence; however, these regions are predicted to be signal peptides in both proteins (Fig. 1, black bar; Interpro). Figure 1 also details predicted HS-binding domains (orange bars) and glycosylation sites (blue diamonds) conserved between Xenopus, human and mouse heparanase 2. Full-length Xenopus, human and mouse heparanase 2 proteins contain the sequence His335_Tyr337 (equivalent to heparanase 1 His296_Tyr298), in which the tyrosine residue has been implicated in HS substrate binding (24). Asn543 in heparanase 2 is also conserved between the three species. This residue has been proposed to be functionally important in HS binding (24) and has undergone a missense mutation in a human UFS family (10). Based on homology with human heparanase 1 and heparanase 2 and using the Interpro database, we identified a region of homology to glycoside hydrolase family 79, the endoglycosidase enzymatic region in heparanase 1. Full-length human heparanase 2 contains certain key features associated with active heparanase 1 (11), e.g. the proton donor Glu258 (Glu243 in heparanase 1) and a putative nucleophile Glu380 (Glu343 in heparanase 1). Heparanase 2 Glu380, however, has a 1 amino acid shift when aligned with heparanase 1 and this may be why human heparanase 2 lacks endo-β-d-glucuronidase activity (17). Interestingly, Xenopus heparanase 2 lacks a Glu residue in this location, suggesting that it too lacks enzymatic activity.

### Heparanase 2 expression during development

**Figure 2.**
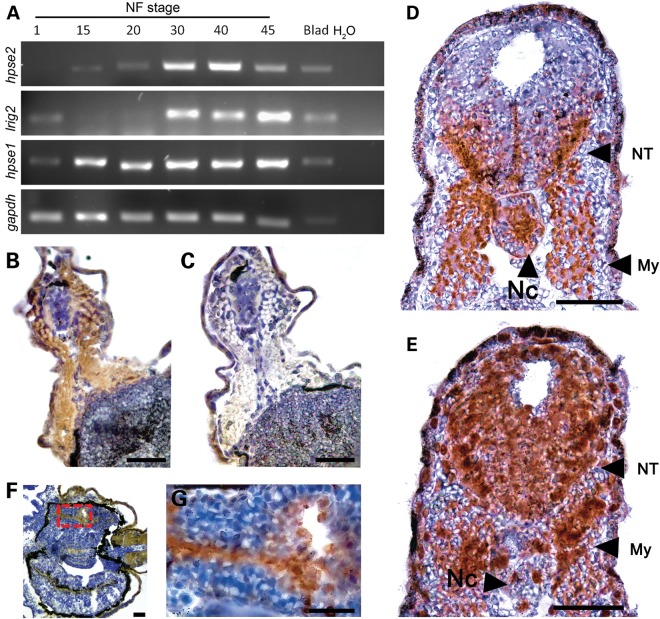
Expression of UFS genes and immunolocalization of UFS proteins. (**A**) RT-PCR analysis of a developmental stage series derived from whole-embryo RNA. *hpse2* was absent at Nieuwkoop–Faber Stage 1 but was detected from Stages 15 to 45. *lrig2* was expressed at Stage 1, then weakly at Stages 15/20, after which transcript levels became prominent. *hpse1* was detected at all stages analysed. All three transcripts were present in adult urinary bladder (Blad). *gapdh* was included as a cDNA loading control. In the H_2_O column, no cDNA was used. (**B**) Transverse section through the trunk of a Stage 40 embryo, with the dorsal surface uppermost. Brown colour shows positive IHC signal for heparanase 2, most prominent in the myotomes flanking the central neural tube, and fainter expression in the ventrolateral neural tube. (**C**) Adjacent section to (B), with the same orientation, showing minimal IHC signal after preincubating primary antibody with the immunizing peptide. (**D**) Transverse section through the trunk at Stage 30, with the dorsal surface uppermost. Prominent heparanase 2 immunoreactivity in detected in the ventrolateral regions of the neural tube (NT), in the notochord (Nc) and in the flanking skeletal muscle myotomes (My). (**E**) Adjacent section to (D), with the same orientation, immunostained for LRIG2. A similar pattern to that of heparanase 2 is observed, extending into the dorsal half of the neural tube. (**F**) Transverse section from a Stage 42 embryo, with the left side uppermost. Three sections of gut are apparent, the archenteron having undergone coiling. Heparanase 2 immunoreactivity is observed in the luminal surface of the gut epithelium, and shown in closer detail in (**G**). (B)–(G) were counterstained with haematoxylin, rendering nuclei blue. Scale bars are 50 µm.

To establish roles for UFS and related genes in embryonic development, we examined expression of *hpse2*, *hpse1* and *lrig2* by reverse transcription-polymerase chain reaction (RT-PCR) in RNA extracted from Xenopus whole embryos from Stages 1 to 45, and from adult urinary bladder (Fig. 2A). We detected transcripts corresponding to maternal (Stage 1) expression of *hpse1* and *lrig2*, but not *hpse2*. At neurulation Stages 15 and 20, corresponding to the formation and development of the neural tube, *hpse2* and *hpse1* were expressed, whereas *lrig2* transcripts were barely detectable. Transcripts for *hpse2*, *hpse1* and *lrig2* were prominent at Stages 30 and 40 when motor nerves which emanate from the truncal neural tube form functional units with adjacent somite-derived myotomes (25,26) and when visceral morphogenesis is underway. Expression of the three transcripts was maintained until Stage 45, the limit of these series. *hpse2*, *hpse1* and *lrig2* were also detected in the urinary bladder of the adult frog.

To identify anatomical regions where UFS proteins are located within the developing embryo, we examined the localization of endogenous heparanase 2 and LRIG2 by immunohistochemistry (IHC). A custom-designed antibody to heparanase 2 was used (Fig. 1, immunizing peptide annotated in green) and the arising immunostaining signals, as ascertained in Stage 40 embryo sections, were abolished by pre-incubation of this antibody with the immunizing peptide (Fig. 2B and C), thus demonstrating signal specificity. As assessed by bright field IHC in Stage 30 embryo sections, heparanase 2 was detected in the neural tube in a ventrolateral pattern excluding the floorplate, and in the notochord and flanking myotomes (Fig. 2D). A similar pattern was found for LRIG2 at Stage 30, with this protein additionally present in the dorsal part of the neural tube but less prominent in the notochord (Fig. 2E). At Stage 40, heparanase 2 immunostaining remained prominent in developing skeletal muscles, but the neural tube signal was less prominent than at Stage 30 (compare Fig. 2B with D). Heparanase 2 was immunodetected in the apical zone/luminal surface of gut epithelium at Stage 42 (Fig. 2E and F), a time point after the archenteron has undergone coiling. These expression data demonstrate the presence of both heparanase 2 and LRIG2 in the developing neural tube and skeletal muscle of the Xenopus embryo, providing circumstantial evidence supporting the hypothesis that a common neuromuscular developmental functional pathway is mediated by these two molecules.

### Dynamic developmental expression of heparanase 2 in the neural tube

**Figure 3.**
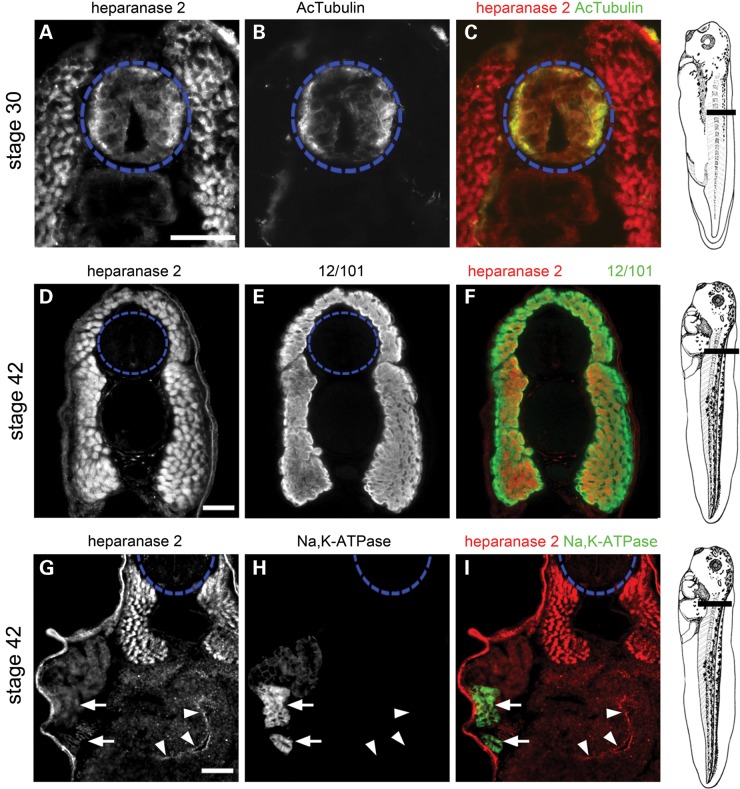
Tissue-specific localization of heparanase 2. Transverse sections through the trunk of Xenopus embryos, as indicated in the diagrams shown on the right, imaged by immunofluorescence, with the neural tube outlined by blue dashed circles. In merged images, heparanase 2 localization is indicated in red while other proteins are indicated in green. (**A–C**) Heparanase 2 colocalized with acetylated α-tubulin (AcTubulin) in the lateral zones of the Stage 30 neural tube. The flanking myotomes were also positive for heparanase 2. (**D–F**) At Stage 42, heparanase 2 was absent in the neural tube but myotomes, co-immunostained with the muscle-marker antibody 12/101 (it is the name of an antibody), remained positive. (**G–I**) The Stage 42 pronephric tubule did not display a specific IHC signal for heparanase 2; note that here, the weak signal is background autofluorescence. The two arrows indicate a proximal tubule which in H and I is seen to be reactive with Na^+^/K^+^-ATPase antibody. The set of three arrowheads in (G)–(I) demonstrate specific heparanase 2 immunostaining in the apical zone of epithelia lining the gut lumen. Scale bars are 50 µm.

To refine the anatomical localization of heparanase 2, co-immunofluorescence was performed between Stages 30 and 42 with antibodies to mark specific cell types. At Stage 30, heparanase 2 immunoreactivity in the ventrolateral neural tube overlapped with that of acetylated α-tubulin (Fig. 3A–C), a cytoskeletal neuronal protein (27), suggesting that heparanase 2 was located in neural cell bodies. By Stage 42, neural tube-specific immunolocalization of heparanase 2 was lost (Fig. 3D and G). At both Stages 30 and 42, heparanase 2 also colocalized in cells positive for 12/101 (it is the name of an antibody) (Fig. 3A and D–F), a marker of skeletal muscle myofibres (28). Given the fact that *HPSE2* mutations in humans can be associated with kidney disease causing renal failure, we also sought colocalization of heparanase 2 with the kidney epithelial marker Na, K-ATPase (29) at Stage 42, when a pronephros has developed on each side of the embryo, each with a duct terminating in the cloaca. No significant IHC signal for heparanase 2 was detected in the pronephric tubule (Fig. 3G and H, arrows) at Stage 42. Similar lack of signal was observed at Stages 32 and 40 (data not shown). At Stage 42, heparanase 2 was immunodetected in the apical gut epithelium (Fig. 3G–I, arrowheads).

### Morpholino-induced knockdown of heparanase 2

**Figure 4.**
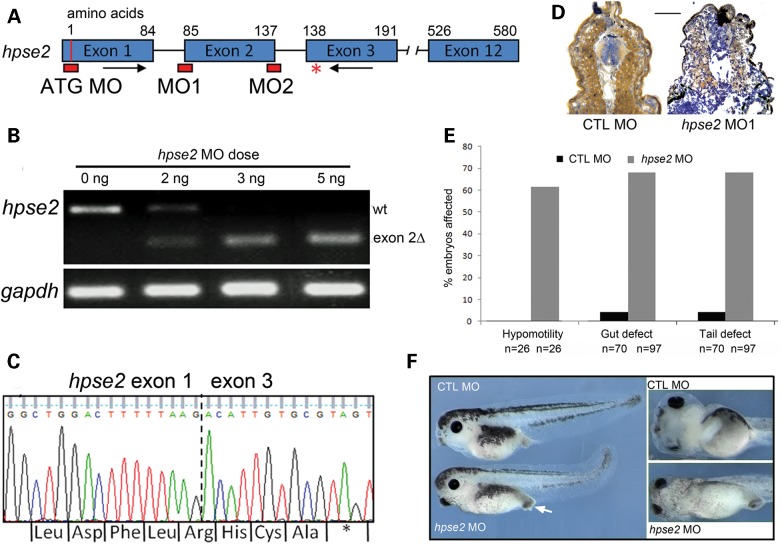
Morpholino knockdown of heparanase 2. (**A**) Schematic diagram of the *X. tropicalis hpse2* gene, showing: exons (blue blocks); ATG MO and splice MO targets (red blocks) at the splice acceptor (MO1) and splice donor (MO2) sites of exon 2; PCR primers flanking exon 2 (black arrows); and the premature stop codon in exon 3 (red asterisk) generated by MO1. (**B**) Injection of increasing amounts of MO1 induced missplicing of *hpse2*, with 5 ng abolishing expression of wild-type (wt) *hpse2* mRNA in favour of a shorter mRNA (exon 2Δ). (**C**) Sanger sequencing of the shorter PCR product confirmed absence of exon 2 (indicated by dotted vertical line). A novel, in-frame pre-mature stop codon was generated in exon 3 (asterisk). (**D**) Injection of 5 ng MO1 led to near-complete loss of heparanase 2 neural tube and myotome immunoreactivity, as demonstrated in this Stage 40 embryo; CTL MO on left and MO1 on right. (**E**) Frequency (%) of the hypomotility, lack of gut looping (gut defect) and tail curvature (tail defect) phenotypes associated with administration of CTL MO or MO1, with total numbers of embryos assessed indicated by ‘*n*’. (**F**) The upper two images depict CTL MO-administered embryos and the lower two images show effects of MO1. Left-hand section depicts embryos viewed from the side; note the protruding proctodeum (white arrow) and tail curvature in the morphant. The two frames on the right depict the embryos viewed from their ventral aspects; note that gut coiling is present in the control embryo but not in the morphant.

We next used a morpholino (MO)-induced knock-down approach to determine the requirement for heparanase 2 during embryo development. We designed three MOs, to target the splice acceptor (MO1) and splice donor (MO2) of exon 2 and the ATG start codon (Fig. 4A, red blocks). Single-cell embryos were injected with increasing doses of MO1 (1–5 ng) and cultured to Stage 36 when *hpse2* messenger RNA (mRNA) from whole embryos was analysed by RT-PCR using primers flanking exon 2 (Fig. 4A arrows and B). In embryos injected with standard control (CTL) morpholino (CTL MO), a band was amplified of a size predicted for wild-type *hpse2* mRNA. Increasing doses of MO1 were associated with amplification of a smaller band, consistent with the size predicted upon exclusion of exon 2 (exon 2Δ in Fig. 4B). At a 5 ng dose of MO1, only the shorter band was amplified. Sequencing showed the longer PCR product was wild-type *hpse2*, while the smaller PCR product corresponded to *hpse2* lacking exon 2, with the resultant mRNA containing a novel, in-frame pre-mature stop codon in exon 3 (Fig. 4C).

To determine the effect of MO1 on heparanase 2 protein, IHC of Stage 40 embryos was undertaken. The 5 ng dose of MO1 led to a near-complete loss of heparanase 2 on immunostaining (Fig. 4D). Embryos injected with 5 ng MO1 were incubated to tadpole stages and three main phenotypes were observed, reproducible over numerous sets of injections. The first phenotype, apparent in approximately two-thirds of MO1-administered embryos, concerned hypomotility (Fig. 4E). Embryos injected with CTL MO hatched normally at Stages 26–28, displaying the appropriate physical reflexes (the ‘hatching reflex’) to facilitate this process, and thereafter responded to touching either the head or the tail by swimming away (the ‘escape reflex’). In contrast, those injected with 5 ng MO1 hatched late, around Stage 35, with embryos continuing to elongate within their enveloping embryonic membrane prior to hatching. Morphants lacked both the hatching reflex and later, the escape reflex. These defects were also noted at the lower MO1 dose of 2.5 ng, which did not generate the gut or tail defects, described below. In embryos with these skeletal muscle motility defects, blood continued to circulate, indicating intact heart muscle function.

**Figure 5.**
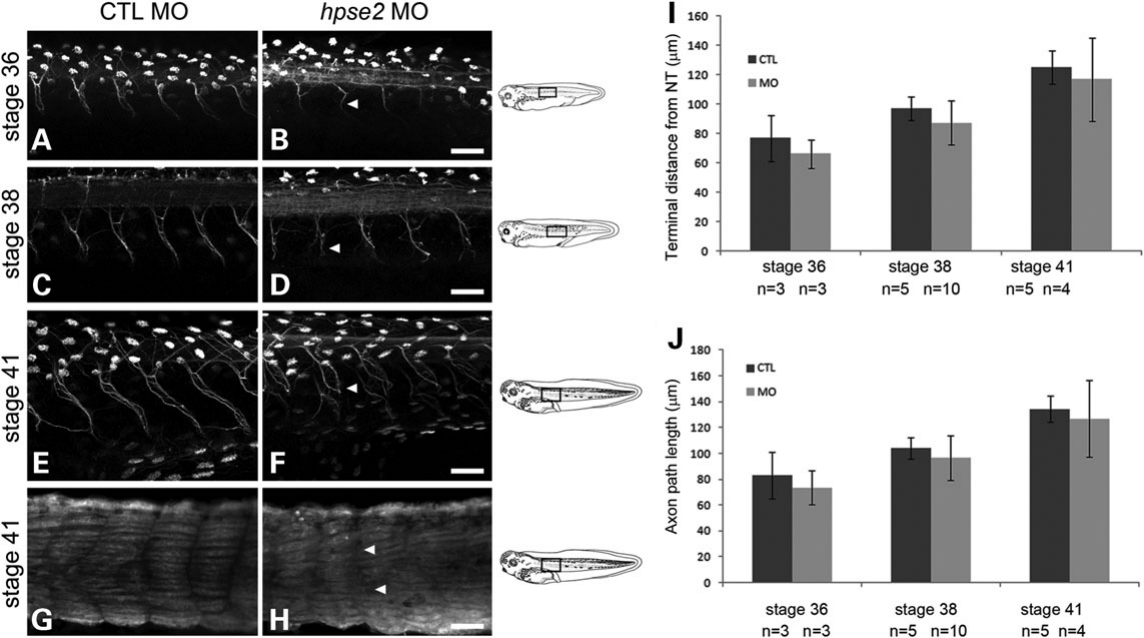
Visualization of motor neurons in parasagittal imaging plane. (**A)**–(**F**) whole-mounts immunostained with antibody to acetylated α-tubulin. This labels axons and also multiciliated round organs in the skin, the latter appearing as white ovals. (**G**) and (**H**) were probed with the muscle antibody 12/101. (A), (C), (E) and (G) are from CTL MO-administered embryos while (B), (D), (F) and (H) are from MO1-administered embryos. Scale bars are 50 μm. (A–F) Across the top of each frame, a longitudinal section of the neural tube is evident, with the anterior to the left. In morphants, at Stages 36, 38 and 41, neurons which had emanated from the neural tube were regularly spaced but their axons lacked compact bundling and coherent directional extension seen in controls. The irregular topography of certain morphant nerves is indicated by arrowheads in (B), (D) and (F). (**G** and **H**) Morphants showed lack of clear separation of skeletal muscle blocks at somitic boundaries (arrowheads in H). (**I** and **J**) Nerve lengths (means ± SD), as assessed by determining the shortest distance between where they exited the neural tube and their overt termini (I) or by tracing individual nerves (J). The average length of 4–6 nerves per embryo was used to generate a value for each embryo, with 3–10 embryos in each experimental group, as indicated.

Secondly, about two-thirds of MO1-administered embryos had a gut which lacked normal coiling; instead the organ remained as an ovoid mass (Fig. 4E and F), as normally found in the undifferentiated archenteron (30). This phenotype was associated with a protruding proctodeum (Fig. 4F) but the shape of the cloaca appeared grossly normal (data not shown). Furthermore, imaging pronephric tubules and ducts did not reveal any gross anatomical anomalies (data not shown) nor were embryos oedematous (Fig. 4F). Thirdly, each embryo with a gut defect also displayed dorsal kinking of its tail (Fig. 4E and F). The gut and tail defects were found in <5% of embryos administered CTL MO, and CTL MO embryos never had motility defects (Fig. 5E). The incidence of each of the three (i.e. motility, gut and tail) phenotypes in the 5 ng MO1 morphants versus embryos administered CTL MO was highly significant (Fisher's exact test, *P* < 0.001). Eleven percent of embryos administered CTL MO and 16% of those administered MO1 showed non-specific effects (data not shown; *P* = 0.38). Injection of 5 ng of MO1 proved lethal beyond Stage 41, possibly as result of the complete failure of gut formation/patterning. The motility, gut and tail defects were replicated upon administration of either MO2 targeting the exon 2 splice donor site or the ATG MO (Fig. 4A; data not shown), supporting the specificity of the MO1 effects.

### Heparanase 2 knockdown alters peripheral nerve and myotome morphology

Given the lack of motility displayed by the majority of MO1-injected embryos, confocal microscopy of whole-mount embryos probed with acetylated α-tubulin antibody was used to seek motor neurons emanating from the neural tube. In MO1 morphants at Stages 36, 38 and 41, motor neurons were present and their spacing along the anterior–posterior axis appeared similar to that found in controls (Fig. 5A–F). In morphants, however, axons within nerve trunks appeared less compactly bundled than those in CTL MO-administered embryos. Moreover, in morphants, the paths of individual nerves meandered compared with the more linear paths in stage-matched controls (e.g. compare A with B, C with D and E with F in Fig. 5). For each embryo up to six nerves emanating from the neural tube were scored as being ‘normal’ or ‘abnormal’, the latter defined by having one or more of the following defects: (i) perpendicular projection from the neural tube, (ii) non-linear path, (iii) lack of coherent axonal bundling and/or (iv) abnormally splayed nerve termini. In Stage 36 embryos, of 22 nerves imaged in CTL MO animals, one had abnormal morphology and of 14 nerves imaged in morphants, 12 were abnormal (Fisher's exact test *P* < 0.0001). In Stage 36 embryos, of 22 nerves imaged in CTL MO animals, two were abnormal and of 30 nerves imaged in morphants, 26 were abnormal (Fisher's exact test *P* < 0.0001). In Stage 40 embryos, of 17 nerves imaged in CTL MO animals, three were abnormal and of 18 nerves imaged in morphants, all were abnormal (Fisher's exact test *P* < 0.0001). Measurements of axonal length (distance between exit from neural tube to detectable termini and axonal path length) revealed a tendency for nerves in MO1-administered embryos to be shorter than in the control ones, although this did not reach statistical significance (Fig. 5I and J). At Stage 41, using the myofibre-specific 12/101 antibody, a lack of clear separation of skeletal muscle fibres at somite boundaries was apparent in morphant versus CTL MO-injected embryos (Fig. 5G and H), suggesting mildly dysmorphic myotome formation.

### Effects of heparanase 2 knockdown on molecules required for neuromuscular development

**Figure 6.**
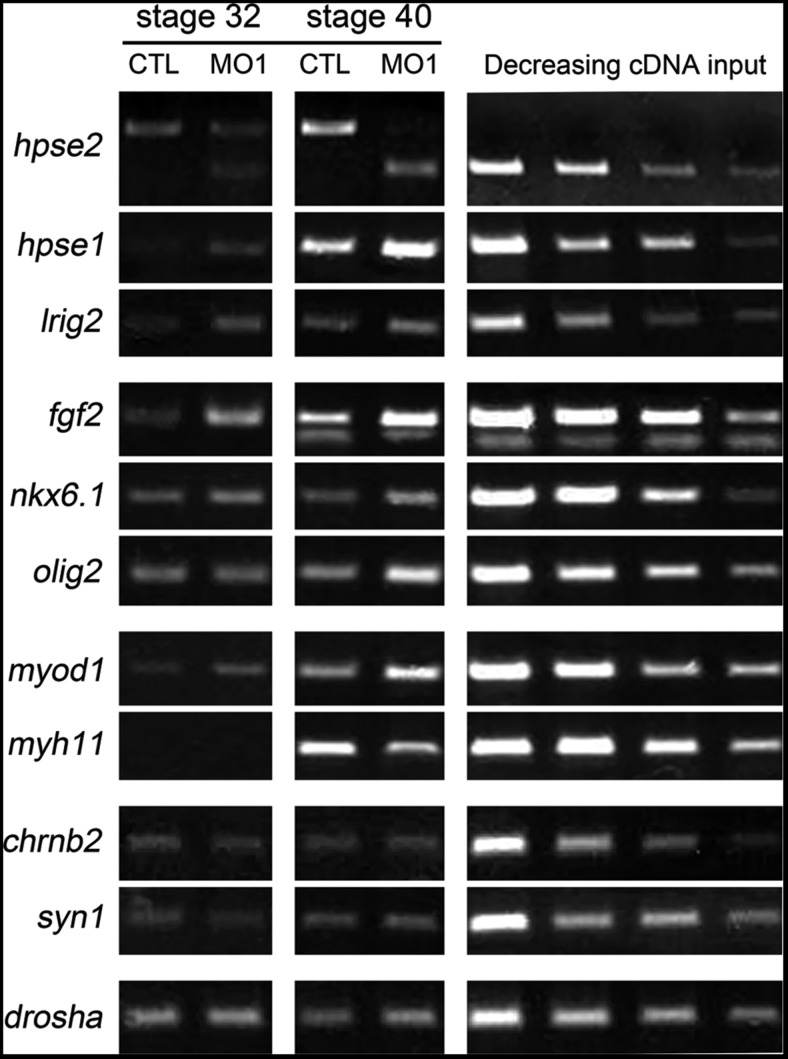
Expression analyses in heparanase 2 knock-down embryos. RNA from pools of Stages 32 and 40 control (CTL MO) and morphant (*hpse2* MO1) embryos was subjected to RT-PCR, with serial dilutions of cDNA depicted on the right of each row. *drosha*, which encodes an RNase III enzyme, was used as a housekeeping control. Morphants had: downregulated wt *hpse2* exon 2 and the appearance of the shorter exon 2Δ amplicon; increased levels of *hpse1* and *lrig2*; increased levels of *fgf2* and of the neuronal precursor markers *olig2* (at Stage 40) and *nkx6.1*; upregulated *myod1*, encoding a skeletal muscle transcription factor, and downregulated *myh11*, encoding a smooth muscle myosin (at Stage 40). Levels of *syn1* and *chrnb2*, encoding synaptic molecules, were similar in morphants and controls.

The above data show that heparanase 2 is required for both normal embryonic motility and the shapes of motor neuron fascicles in developing Xenopus, and that heparanase 2 protein is present in the ventrolateral neural tube where motor neurons originate. Therefore, we next analysed the expression of key molecules required in the ventrolateral neural tube for motor neuron formation. Given that heparanase 2 was immunolocalized in myotomes, we also assayed markers of muscle differentiation and synaptic molecules. Semiquantitative RT-PCR was undertaken using RNA from pools of CTL MO-injected embryos and heparanase 2 morphants. Figure 6 depicts results from two stages, Stages 32 and 40; however, similar results were found in further experiments at Stage 39 and a Stage 40 repeat experiment. At each stage, both the control and the morphant cDNA were generated from a pool of three embryos. As expected, using exon 2 flanking primers, *hpse2* transcripts were markedly reduced in MO1-administered embryos, with a shorter PCR product lacking exon 2 generated. Of note, levels of *hpse1* were increased in morphants at both stages. In addition, *lrig2* transcripts appeared modestly upregulated in morphants. Morphants showed modest increases in levels of *fgf2*, *olig2* and *nkx6.1*, transcripts which encode molecules functional in motor nerve precursor cells (31–34). Levels of *myod1*, which encodes a skeletal muscle transcription factor (35,36), were increased in morphants at Stages 32 and 40. In contrast, levels of *myh11*, encoding a smooth muscle cytoskeletal protein (37), were reduced in heparanase 2 morphants at Stage 40 when *myh1* transcripts became detectable in CTL MO embryos. The expression of the synaptic markers *syn1* encoding synapsin, a synaptic vesicle protein (38) and *chrnb2*, encoding a subunit of the nicotinic acetylcholine receptor (39) were similar in the heparanase 2 knock-down embryos compared with stage-matched CTL embryos.

### An inverse relationship between heparanase 2 and pERK

**Figure 7.**
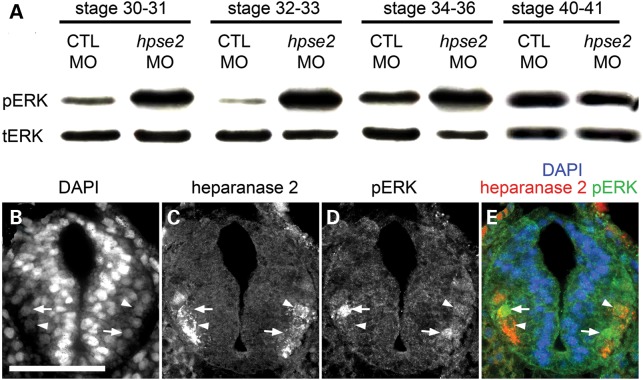
pERK detected by western blotting and IHC. (**A**) Western blots for pERK and tERK in sets of pooled embryos collected, as indicated, between Stages 30 and 41. Note that the signal for pERK was clearly increased in heparanase 2 knock-down embryos versus CTL MO embryos between Stages 30 and 36. This effect was no longer apparent at Stages 40 and 41 (**B–E**) Transverse section of the neural tube of Stage 32 uninjected embryo, with the dorsal surface uppermost, showing (B) all nuclei stained with diamindino-2-phenylindole (DAPI), (C) heparanase 2 immunoreactivity, (D) pERK immunoreactivity, and (E) merged image. pERK+ nuclei (two are indicated by arrows) and heparanase 2+ cells (two are indicated by arrowheads) are detected in the lateral zones of the tube. Note that the cells with strong nuclear pERK immunostaining and those with strong heparanase 2 signals tend to be mutually exclusive. The image is representative of experiments on three embryos.

Given that (i) heparanase 2 inhibits the activity of heparanase 1 (17), (ii) heparanase 1 enhances FGF2 bioavailability and its signalling via pERK (14–16), (iii) FGF signalling is implicated in neuromuscular development (40,41) and (iv) transcripts encoding heparanase 1 and FGF2 were elevated in heparanase 2 morphants, we next analysed pERK levels in heparanase 2 morphants versus controls and compared tissue expression patterns of pERK and heparanase 2 in wild-type embryos. Protein was extracted from pools of whole embryos (three for each blot) harvested at different stages and both pERK and total ERK (tERK) immunoreactive proteins sought by western blotting (Fig. 7A). At Stages 30/31, 32/33 and 34/36, semiquantification of signals showed that the ratio of pERK to tERK was increased in heparanase 2 knock-down embryos compared with CTL MO-administered embryos. In contrast, the pERK/tERK ratio was similar in the morphant and control groups at Stage 40/41. Separate sets of control and morphant embryos were then analysed (data not shown); two further sets at Stage 32/33 confirmed that heparanase 2 knockdown was associated with increased pERK/tERK, and another set at 40/41 showed similar levels between the two groups. Collectively, the results show a constant pattern of upregulation of pERK signalling between Stages 30 and 36, when truncal motor nerves are normally becoming functionally active, correlating with increasing embryonic motility.

In uninjected embryos, both heparanase 2 and pERK IHC signals were prominent in cells in the ventrolateral aspects of the Stage 32 neural tube (Fig. 7B, representative of three experiments), the regions where motor neurons are specified and from which their axons emerge. Interestingly, when considering individual cells, those with intense pERK nuclear signals appeared to have little heparanase 2 immunoreactivity (see the pERK/heparanase 2 merged image in Fig. 7E). These descriptive observations, coupled with the upregulation of pERK in heparanase 2 knock-down embryos, support the hypothesis that, in normal development, heparanase 2 downregulates pERK in the ventrolateral neural tube.

## DISCUSSION

To our knowledge, this study is the first to demonstrate *in vivo* developmental roles for heparanase 2 in any model organism and is also the first to shed light on the pathogenic biological mechanisms underpinning the clinical features of UFS. Specifically, in *X. tropicalis*, we demonstrated that heparanase 2 is required for functional peripheral neural development and that it modulates growth factor signalling during embryogenesis. In turn, these results support the contention that congenital peripheral neuropathy is a key feature of UFS.

By bioinformatic analyses, we identified the Xenopus orthologue of human heparanase 2. The protein is extensively conserved between frog, mouse and human, consistent with conserved functions between diverse species. Although we identified a domain in Xenopus heparanase 2 with homology to the glycosyl hydrolase domain of heparanase 1 (also present in human heparanase 2), the substitution of key residues required for degradation of HSPGs suggests that, like human heparanase 2 (17), Xenopus heparanase 2 lacks this enzymatic activity.

In biochemical assays, heparanase 2 can act as an inhibitor of heparanase 1 enzymatic activity (17). We therefore examined the temporal expression of *hpse* genes in developing Xenopus embryos. *hpse2* transcripts were not present at Stage 1 but were detectable at Stages 15 and 20, and became prominent at Stages 30 and 40. *hpse1* transcripts were maternally expressed and robust levels were detected at all time points assessed between Stages 15 and 45, the limit of the study. We demonstrated that, at Stage 30, heparanase 2 localizes to the ventrolateral neural tube. Although we did not perform IHC for heparanase 1, immunostaining for this protein in Xenopus embryos in neural tube, somites and gut at Stage 39 has been shown (42). Thus, the potential for a regulatory role for heparanase 2 on heparanase 1 activity exists from neurulation stages onwards, within the neural tube. The more prominent early expression of *hpse1* in contrast with *hpse2* suggests that heparanase 1 has independent functions in the early embryo. This is supported by the fact that heparanase 1 knockdown in Xenopus is lethal between Stages 17 and 28 (43), a developmental time period which corresponds to neural tube formation, and our observation that heparanase 2 knockdown resulted in lethality much later in development (Stage 41). Finally, we observed that knockdown of heparanase 2 results in increased levels of *hpse1* transcripts. Hence, a normal function of heparanase 2 may be directly or indirectly downregulate the transcription of *hpse1*. Such an effect would provide a second molecular ‘brake’ on heparanase 1 activity in addition to the already established direct biochemical blockade on this protein conferred by heparanase 2 (17).

Heparanase 2 knock-down Xenopus embryos lacked hatching and escape reflexes. Such morphants possessed motor nerves, which emanated from the truncal neural tube at regular intervals as normal. Moreover, although the average length of these nerves running along the inside surface of the myotome was shorter in morphants than in controls, the difference did not reach statistical significance. Conversely, it was clear that motor nerve paths were more meandering in morphants and that the axons in these nerves were less compactly bundled than in control embryos. These results demonstrate that heparanase 2 is required for motor neuron development and/or function.

**Figure 8.**
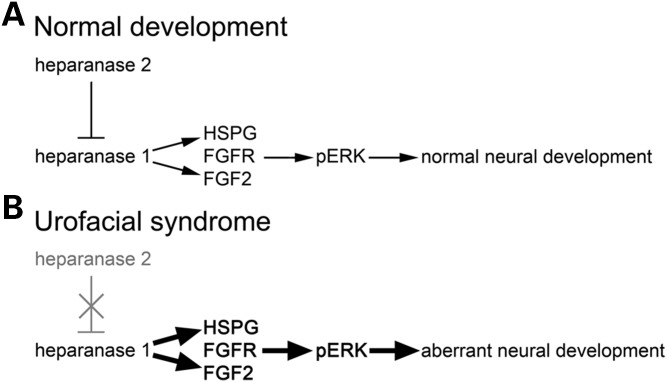
Schematic diagram of proposed heparanase 2 function in FGF signalling and motor neuron development. (**A**) In normal development, heparanase 2 inhibits the activity of heparanase 1, both biochemically by sequestering HSPGs (17) and possibly transcriptionally (this study). This inhibition maintains normal levels of FGF signalling and downstream genes within the embryo, allowing correct motor neuron functional development. (**B**) Loss of heparanase 2 causes upregulation of FGF signalling (and subsequent deregulation of downstream pathway components), perturbing expression of genes required for motor neuron development.

In embryonic frogs, the outgrowth of motor axons is mediated by brain-derived neurotrophic factor but their functional maturation, involving lateral filopodia formation and synapsing with nascent skeletal muscle cells, is modulated by FGF2 derived from myotome cells (40,41). Heparanase 2 is present in the ventrolateral neural tube of Xenopus embryos (this study), the zone containing cell bodies whose motor neuron axons are becoming functional (25,26). In addition, somite-derived myotomes contain heparanase 2. We therefore analysed FGF2 expression and activity, using the surrogate marker of pERK/tERK ratio, in heparanase 2 morphants. Heparanase 2 knockdown led to increased levels of *fgf2* transcripts at Stages 32 and 40. We also detected pERK+ nuclei in the lateral zones of the neural tube, overlapping heparanase 2 protein, and the pERK/tERK ratio was markedly increased in heparanase 2 morphants between Stages 31 and 36. FGF2 is also implicated in the induction of *olig2* (44), a transcription factor that defines the motor neuron progenitor domain of the ventrolateral neural tube, from where motor neurons and oligodendrocytes are sequentially generated (31); we identified, at Stage 40, a modest upregulation of *olig2*. Moreover, *nkx6.1*, encoding another transcription factor functional in motor nerve precursors (34), was also increased at Stage 40. Hence, downregulation of heparanase 2 leads to an upregulation of FGF2 bioactivity. We speculate that this upregulation of FGF2 bioactivity in response to reduced heparanase 2 proceeds indirectly via heparanase 1, given that heparanase 1 has established effects on FGF2 signalling and levels of pERK (14–16). Putting all the historic and current experimental observations together, we conclude that a normal embryonic function of heparanase 2 is to buffer growth factor signalling, including that mediated by FGF2 (Fig. 8A). When this signalling system is abnormally upregulated (Fig. 8B), motor neuron functional development is perturbed resulting in skeletal muscle motility defects.

Alongside mutations in *HPSE2*, UFS is also caused by mutations in *LRIG2*. The most studied member of the LRIG family is LRIG1, which downregulates growth factor RTK signalling (20,21). While less is known about LRIG2, within the mouse, Lrig2 expression is enhanced in the neurons and sensory epithelia of the inner ear and *Lrig2* homozygous null mice display an attenuated neuronal response to sound (45). LRIG2 is permissive for oligodendroglial and glioblastoma-like brain tumour formation in a mouse model (46) and downregulation of LRIG2 in cultured glioma cells led to ligand-mediated loss of the epidermal growth factor receptor (an RTK), cell cycle arrest and increased apoptosis (22).

We identified overlapping expression patterns of heparanase 2 and LRIG2 in embryonic frog neural tube and myotomes, consistent with the hypothesis that the proteins are involved in similar biological molecular pathways, as is the fact that biallelic mutation of either gene causes an identical disease in human UFS (4). Knockdown of heparanase 2 caused a small increase in levels of *lrig2* in whole embryos. Thus, it is possible that the two genes/proteins are involved in a feedback loop or can interact in a redundancy mechanism. It is currently unknown whether a reciprocal relationship exists whereby alterations in LRIG2 expression lead to changes in expression of *hpse2*. In terms of UFS associated with *HPSE2* mutations, it is possible that pathogenic effects are mediated by LRIG2 dysregulation and studies to test the ability of LRIG2 overexpression/knockdown to rescue the heparanase 2 morphant phenotype in Xenopus can be undertaken in future. We note that a previous study detected *lrig2* transcripts enriched in the dorsal organizer during Xenopus gastrulation, with ectopic *lrig2* expression induced in ventral regions upon inhibition of bone morphogenetic protein signalling (47). These experiments are consistent with LRIG2 protein having a role in primary neural differentiation, although the process of primary neurulation is clearly grossly intact in humans with UFS who carry biallelic, postulated null, *LRIG2* mutations (18).

What are the implications of the current Xenopus study for our understanding of the human UFS disease? It has been speculated that, based on the clinical features, the human syndrome has a neurological basis or bases (2,3) but, thus far, hard evidence has been lacking. Extrapolating the data presented in the current study, demonstrating a requirement for heparanase 2 for correct peripheral motor neuron functional development in the trunk region, we propose a similar requirement for heparanase 2 in the development of other peripheral motor nerves, including those of the human face and viscera. These would clearly include the facial nerve, corresponding to the abnormal facial movements and weakness (5,6) observed in individuals with UFS. With regard to the bladder and bowel evacuation dysfunction which also feature in the syndrome, consideration must be given to two sets of motor neurons, i.e. somatic pudendal nerves which stimulate constriction of the external sphincters of the bladder and gut, and autonomic nerves which modulate contraction of detrusor and gut smooth muscle (48). In fact, both heparanase 2 and LRIG2 have been immunodetected in presumed autonomic nerve trunks in human bladders (18), but whether these proteins are also present in external sphincter nerves is unknown. Notably, a human precedent has already been set with respect to a genetic autonomic neuropathy generating congenital bladder dysfunction because mutation of *CMRM3*, encoding the M3 acetylcholine receptor on detrusor smooth muscle, leads to such a disease (49). Interestingly, pERK has already been implicated in the neural control of mouse bladders. Levels of pERK are upregulated in the L6 spinal cord segment after spinal cord injury in the rat, and concomitant with abnormally high frequencies of bladder contraction; intrathecal administration of PD98059, a specific inhibitor of ERK phosphorylation, reduces the frequency and amplitude of abnormal bladder contractions (50). Furthermore, injury-induced upregulation of pERK causes increased frequency.

The embryonic frog is not an appropriate model in which to study mammalian bladder or hindgut dysfunction because the just-formed cloaca simply acts as a passive conduit for excretory products such as pronephric urine. We did, however, note that heparanase 2 is localized to the apical zones of embryonic gut epithelia and that heparanase 2 morphant embryos displayed a lack of normal gut rotation. Heparanase 2 morphants had decreased levels of transcripts encoding a smooth muscle myosin, *myh11*. Intriguingly, we found that levels of *myod1*, encoding a skeletal muscle transcription factor, were increased in heparanase 2 morphants; this may indicate that the myotome, which also normally contains heparanase 2 and which is mildly dysmorphic in heparanase 2 morphants, is also defective when heparanase 2 is lacking. These Xenopus observations point to defects of both classes of muscle and, by analogy, lead to a novel suggestion that, in addition to neural defects, there may also exist aberrations of smooth and skeletal muscle which help to explain the spectrum of clinical disease in UFS.

The severe kidney disease present in some individuals with UFS (2,4) may be the result of post-natal damage caused, for example, by ascending bacterial pyelonephritis, a well-documented complication in a subset of patients. However, an alternative, non-exclusive, hypothesis is that some UFS kidney disease represents congenital defects, i.e. aberrant kidney development before birth. A major role of the embryonic amphibian pronephros is to excrete excess water which enters the body via the skin and by ingestion (51), and thus kidney failure would manifest as oedema. In the current study, heparanase 2 morphant embryonic frogs were not oedematous and they contained pronephric tubules and ducts which did not appear to be malformed. Both observations suggest that heparanase 2 does not have critical roles in structural and functional differentiation of the embryonic pronephros, although subtle effects cannot be excluded. An implication could be that kidney disease in humans with UFS does not have a developmental origin. However, caution is required in such a conclusion because the mammalian adult kidney forms from the metanephric embryonic organ rather than a pronephros.

## MATERIALS AND METHODS

### Embryo culture and microinjection

Animal experiments were approved by the University's Ethical Review Panel and were undertaken under UK Home Office project licence PPL 40/3550. *Xenopus tropicalis* embryos were obtained and cultured by standard methods (52) and staged according to Nieuwkoop and Faber (53). Morpholino injections were performed using a Picospritzer III microinjector (Intracel) to inject 1 nl volumes at the one-cell stage. During injection and for subsequent incubation, embryos were maintained in 0.1× Marc's modified Ringer's (MMR)/3% Ficoll at 22°C on agarose-bottomed wells. Prior to gastrulation, embryos were removed to 0.1× MMR. At Stage 17, embryos were removed to 0.1× MMR at 24–26°C in plastic wells for the remainder of culture. Tail defects and loss of movement/escape responses were scored from Stage 30 and gut coiling defects from Stage 40. Where required, embryos were fixed in MEMFA (0.1 M MOPS [3-(*N*-morpholino) propanesulphonic acid], 2 mM EGTA [ethylene glycol tetraacetic acid], 1 mM magnesium sulphate, 3.7% formaldehyde), dehydrated into 100% methanol and stored at −20°C.

### Morpholino oligonucleotides

Antisense MO oligonucleotides (MOs) and standard CTL MO were designed by and purchased from Gene Tools LLC. Three antisense MOs were designed to target the acceptor (MO1) and the donor (MO2) site of Xenopus *hpse2* and to target the ATG start codon. The MO1 MO sequence was TTGGAACTGCATGTAAAGAGAGACA; MO2 and ATG MO sequences are available on request. MOs were resuspended in diethyl pyrocarbonate (DEPC)-treated dH_2_O to a stock concentration of 20 ng nl^−1^.

### RT-PCR analysis of gene expression

Total RNA was extracted from pools of five embryos (or up to 50 mg of tissue) using an RNeasy Mini Kit (Qiagen) and resuspended in non-DEPC-treated RNase-free dH_2_O (Life Technologies). RNA (1 µg) was reverse transcribed using the High Capacity RNA-to-cDNA Kit (Life Technologies) in 20 µl reactions. PCR reactions comprised 1× GoTaqGreen MasterMix (Promega), up to 1 µl cDNA reaction following equalizing of sample housekeeper levels and 500 nm each primer (Eurofins) and were performed on a Veriti 96-well fast thermal cycler (Life Technologies) using the following basic cycling conditions: 95°C 2 min, then (94°C 30 s/annealing temperature 30 s/72°C 30 s) × cycle number, then 72°C 5 min.

PCR amplifications were carried out using the following primer oligonucleotides, with annealing temperature/cycle number indicated in brackets:

***hpse2*** (standard RT-PCR; Fig. 2) f CTGAAGAATCCGGCAAAGAG r CTACGCACAATGTCGTCCTC (60°C/30); ***hpse2*** (spanning exon 2; Figs 4 and 6) f TTAAGTTCCAAGAGGTTAGTGAC r ATGAAACCATCTAGAAGGGCT (58°C/30); ***hpse1*** f TTTGGCGCAGGATCCTATAA r CCAGTAATCTGGTAAGGGTTCAA (60°C/33); ***lrig2*** f AGCTCTTGGATCTAGACTTGTCAT r GCCCTTTAAAGACACCTTCAGC (60°C/27); ***fgf2*** f ACTGCAAGAATGGGGGCTAC r GGCAAGGTAGCGATTTGCAG (60°C/30); ***nkx6.1*** f GTAATGCAGGGCTCACCTTG r CCAGAAAAGGTTGGTCGGGA (60°C/30); ***olig2***f CTCCCATCCATCTTCCCACG r GCACTTGGCACATACTGCAC (65°C/30); ***myod1*** f GCAACGCGATTCGCTACATA r GTCTGTCATGCCATCGGAGCAG (65°C/28); ***myh11*** f ACAGGGTCAGATGAAAGACTTC r AGCCTCCAAACTCTTGGCTT (60°C/28); ***chrnb2*** f TGACTCAGGAGTGGGAGGAT r GACACCTCGTACATCCCGTC (60°C/30); ***syn1*** f TTGTGAGGTCACTCAAGCCG r ATAGACGGAGTGCAGGGAGT (60°C/30); ***drosha*** f TTACAGACCGCTGTTTGCTG r CAATTCGAGAGGGAGTTTCG (60°C/29).

### Antibodies and western blotting

A custom rabbit anti-heparanase 2 antibody was designed and manufactured (Generon). The immunizing peptide sequence used was QLDPSIIHDGWLDC (Fig. 1). Commercially available antibodies used in this study were: mouse anti-acetylated tubulin (Sigma), mouse anti-12/101 (Developmental Studies Hybridoma Bank), rabbit anti-ERK (Cell Signalling), mouse anti-pERK (Sigma–Aldrich), mouse anti-Na,K-ATPase (Abcam), rabbit anti-LRIG2 (Abgent), horseradish peroxidase (HRP)-coupled anti-mouse and anti-rabbit (Life Technologies). To test specificity, anti-heparanase 2 antibody was incubated overnight at 4°C on a rotator with a 50-fold excess immunizing peptide or a control reaction of 50-fold excess non-specific peptide, prior to use in IHC applications. Western blotting was performed by standard methods. Total protein was extracted from pools of three embryos, with amounts equivalent to one embryo separated via SDS–PAGE.

### Embryo sectioning and immunostaining

For sectioning, MEMFA-fixed embryos were rehydrated overnight in H_2_O/25% fish gelatine (Sigma), then placed in moulds containing fresh H_2_O/25% fish gelatine. Blocks were solidified on dry ice and sectioned in a cryostat (Leica) at 10 µm. Slides were dried for 1h in a fume hood, cleared in acetone for 2min then rinsed in phosphate buffered saline (PBS)/0.1% Triton X-100 (PBST). Where required, slides were stored at −20°C for later use. Sections were blocked by incubating for 30 min at room temperature with PBST/5% heat-treated lamb serum (HTLS; Life Technologies). Primary antibodies were made up in PBST/5% HTLS and applied overnight at 4°C. For bright field IHC, biotinylated secondary antibodies were applied for 2h at 4°C, washed in PBS then incubated for 1h at 4°C with streptavidin-HRP (Vector). Slides were incubated with diaminobenzidine (DAB) (Vector) for 2.5 min, stained with haemotoxylin for 30 s, mounted with Mowiol (Sigma–Aldrich) then a coverslip applied and sealed with nail varnish. For immunofluorescence, Alexa Fluor secondary antibodies (Life Technologies) were applied for 1 h at room temperature, washed in PBS and incubated with diamindino-2-phenylindole (DAPI) (Vector) for 10min, washed in PBS, mounted with Mowiol then a coverslip applied and sealed with nail varnish.

### Whole-mount immunofluorescence and nerve length calculations

Whole-mount immunofluorescence was performed by standard methods (27) and imaged in Murray's solution (2 : 1 benzyl benzoate : benzyl alcohol) on an Olympus laser confocal microscope. Images from the confocal microscope were merged and flattened in ImageJ. Neuron lengths were calculated using the ImageJ line tool, measuring in a straight line from the neural tube to the tip of the nerve to give terminal distance or following the route of the nerve to give the axon path length.

### Online resources

Interpro (www.ebi.ac.uk/interpro, last accessed on 10 April 2014); Metazome (www.metazome.net, last accessed on 10 April 2014); Online Mendelian Inheritance in Man (OMIM; www.ncbi.nlm.nih.gov/omim, last accessed on 10 April 2014); Xenbase (www.xenbase.org ©Pieter D. Nieuwkoop and J. Faber, 1994); and *Xenopus tropicalis* genome project (genome.jgi-psf.org, last accessed on 10 April 2014).

## FUNDING

This work was supported by Kidney Research UK (RP33/2010) and the Medical Research Council (MR/L002744/1). H.M.S. is a Wellcome Trust Clinical Research Fellow. E.N.H. is the recipient of a Stepping Stone Award from the University of Manchester. Funding to pay the Open Access publication charges for this article was provided by the University of Manchester Library.

## References

[1] Elejalde B.R. (1979). Genetic and diagnostic considerations in three families with abnormalities of facial expression and congenital urinary obstruction: ‘the Ochoa syndrome. Am. J. Med. Genet..

[2] Ochoa B. (2004). Can a congenital dysfunctional bladder be diagnosed from a smile?. Pediatr. Nephrol..

[3] Ganesan I., Thomas T. (2011). More than meets the smile: facial muscle expression in children with Ochoa syndrome. Med. J. Malaysia.

[4] Newman W.G., Woolf A.S., Stuart H.M., Pagon R.A. (2013). Urofacial syndrome. GeneReviews™.

[5] Mermerkaya M., Süer E., Oztürk E., Gülpınar O., Gökçe M.I., Yalçındağ F.N., Soygür T., Burgu B. (2013). Nocturnal lagophthalmos in children with urofacial syndrome (Ochoa): a novel sign. Eur. J. Pediatr..

[6] Garcia-Minaur S., Oliver F., Yanez J.M., Soriano J.R., Quinn F., Reardon W. (2001). Three new European cases of urofacial (Ochoa) syndrome. Clin. Dysmorphol..

[7] Daly S.B., Urquhart J.E., Hilton E., McKenzie E.A., Kammerer R.A., Lewis M., Kerr B., Stuart H., Donnai D., Long D.A. (2010). Mutations in HPSE2 cause urofacial syndrome. Am. J. Hum. Genet..

[8] Pang J., Zhang S., Yang P., Hawkins-Lee B., Zhong J., Zhang Y., Ochoa B., Agundez J.A.G., Voelckel M.A., Gu W. (2010). Loss-of-function mutations in HPSE2 cause the autosomal recessive urofacial syndrome. Am. J. Hum. Genet..

[9] Al Badr W., Al Bader S., Otto E., Hildebrandt F., Ackley T., Peng W., Xu J., Li J., Owens K.M., Bloom D. (2011). Exome capture and massively parallel sequencing identifies a novel HPSE2 mutation in a Saudi Arabian child with Ochoa (urofacial) syndrome. J. Pediatr. Urol..

[10] Mahmood S., Beetz C., Tahir M.M., Imran M., Mumtaz R., Bassmann I., Jahic A., Malik M., Nürnberg G., Hassan S.A. (2012). First HPSE2 missense mutation in urofacial syndrome. Clin. Genet..

[11] McKenzie E., Tyson K., Stamps A., Smith P., Turner P., Barry R., Hircock M., Patel S., Barry E., Stubberfield C. (2000). Cloning and expression profiling of Hpa2, a novel mammalian heparanase family member. Biochem. Biophys. Res. Commun..

[12] Levy-Adam F., Ilan N., Vlodavsky I. (2010). Tumorigenic and adhesive properties of heparanase. Semin. Cancer Biol..

[13] Arvatz G., Shafat I., Levy-Adam F., Ilan N., Vlodavsky I. (2011). The heparanase system and tumor metastasis: is heparanase the seed and soil?. Cancer Metastasis Rev..

[14] Vlodavsky I., Fuks Z., Ishai-Michaeli R., Bashkin P., Levi E., Korner G., Bar-Shavit R., Klagsbrun M. (1991). Extracellular matrix-resident basic fibroblast growth factor: implication for the control of angiogenesis. J. Cell. Biochem..

[15] Myler H.A., West J.L. (2002). Heparanase and platelet factor-4 induce smooth muscle cell proliferation and migration via bFGF release from the ECM. J. Biochem..

[16] Reiland J., Kempf D., Roy M., Denkins Y., Marchetti D. (2006). FGF2 binding, signaling, and angiogenesis are modulated by heparanase in metastatic melanoma cells. Neoplasia.

[17] Levy-Adam F., Feld S., Cohen-Kaplan V., Steingaus A., Gross M., Arvatz G., Naroditsy I., Ilan N., Doweck I., Vlodavsky I. (2010). Heparanase 2 interacts with heparan sulfate with high affinity and inhibits heparanase activity. J. Biol. Chem..

[18] Stuart H.M., Roberts N.A., Bergu B., Daly S.B., Urquhart J.E., Bhaskar S., Dickerson J., Mermerkaya M., Silay M.S., Lewis M.A. (2013). LRIG2 mutations cause urofacial syndrome. Am. J. Hum. Genet..

[19] Guo D., Holmlund C., Henriksson R., Hedman H. (2004). The LRIG gene family has three vertebrate paralogs widely expressed in human and mouse tissues and a homolog in Ascidiacea. Genomics.

[20] Wong V.W., Stange D.E., Page M.E., Buczacki S., Wabik A., Itami S., van de Wetering M., Poulsom R., Wright N.A., Trotter M.W. (2012). Lrig1 controls intestinal stem-cell homeostasis by negative regulation of ErbB signalling. Nat. Cell Biol..

[21] Rafidi H., Mercado F., Astudillo M., Fry W.H., Saldana M., Carraway K.L., Sweeney C. (2013). Leucine-rich repeat and immunoglobulin domain-containing protein-1 (Lrig1) negative regulatory action toward ErbB receptor tyrosine kinases is opposed by leucine-rich repeat and immunoglobulin domain-containing protein 3 (Lrig3). J. Biol. Chem..

[22] Wang B., Han L., Chen R., Cai M., Han F., Lei T., Guo D. (2009). Downregulation of LRIG2 expression by RNA interference inhibits glioblastoma cell (GL15) growth, causes cell cycle redistribution, increases cell apoptosis and enhances cell adhesion and invasion in vitro. Cancer Biol. Ther..

[23] Woolf A.S., Stuart H.M., Roberts N.A., McKenzie E.A., Hilton E.N., Newman W.G. (2014). Urofacial syndrome: a genetic and congenital disease of aberrant urinary bladder innervation. Pediatr. Nephrol..

[24] Gandhi N.S., Freeman C., Parish C.R., Mancera R.L. (2012). Computational analyses of the catalytic and heparin-binding sites and their interactions with glycosaminoglycans in glycoside hydrolase family 79 endo-β-D-glucuronidase (heparanase). Glycobiology.

[25] van Mier P., Armstrong J., Roberts A. (1989). Development of early swimming in Xenopus laevis embryos: myotomal musculature, its innervation and activation. Neuroscience.

[26] van Mier P., ten Donkelaar H.J. (1989). Structural and functional properties of reticulospinal neurons in the early-swimming stage Xenopus embryo. J. Neurosci..

[27] Huang J.K., Dorey K., Ishibashi S., Amaya E. (2007). BDNF promotes target innervation of Xenopus mandibular trigeminal axons in vivo. BMC Dev. Biol..

[28] Dali L., Gustin J., Perry K., Domingo C.R. (2002). Signals that instruct somite and myotome formation persist in Xenopus laevis early tailbud stage embryos. Cells Tissues Organs.

[29] McCoy K.E., Zhou X., Vize P.D. (2008). Collectrin/tmem27 is expressed at high levels in all segments of the developing Xenopus pronephric nephron and in the Wolffian duct. Gene Expr. Patterns.

[30] Tseng H.T., Shah R., Jamrich M. (2004). Function and regulation of FoxF1 during Xenopus gut development. Development.

[31] Zhou Q., Anderson D.J. (2002). The bHLH transcription factors OLIG2 and OLIG1 couple neuronal and glial subtype specification. Cell.

[32] Bronchain O.J., Pollet N., Ymlahi-Ouazzani Q., Dhorne-Pollet S., Helbling J.C., Lecarpentier J.E., Percheron K., Wegnez M. (2007). The olig family: phylogenetic analysis and early gene expression in Xenopus tropicalis. Dev. Genes Evol..

[33] Ribes V., Briscoe J. (2009). Establishing and interpreting graded Sonic Hedgehog signaling during vertebrate neural tube patterning: the role of negative feedback. Cold Spring Harb. Perspect. Biol..

[34] Dichmann D.S., Harland R.M. (2011). Nkx6 genes pattern the frog neural plate and Nkx6.1 is necessary for motoneuron axon projection. Dev. Biol..

[35] Rudnicki M.A., Le Grand F., McKinnell I., Kuang S. (2008). The molecular regulation of muscle stem cell function. Cold Spring Harb. Symp. Quant. Biol..

[36] Havis E., Coumailleau P., Bonnet A., Bismuth K., Bonnin M.A., Johnson R., Fan C.M., Relaix F., Shi D.L., Duprez D. (2012). Sim2 prevents entry into the myogenic program by repressing MyoD transcription during limb embryonic myogenesis. Development.

[37] Takeda Y., Koh S.D., Sanders K.M., Ward S.M. (2008). Differential expression of ionic conductances in interstitial cells of Cajal in the murine gastric antrum. J. Physiol..

[38] Lu B., Czernik A.J., Popov S., Wang T., Poo M.M., Greengard P. (1996). Expression of synapsin I correlates with maturation of the neuromuscular synapse. Neuroscience.

[39] Baldwin T.J., Yoshihara C.M., Blackmer K., Kintner C.R., Burden S.J. (1988). Regulation of acetylcholine receptor transcript expression during development in Xenopus laevis. J. Cell Biol..

[40] Li P.P., Chen C., Lee C.W., Madhavan R., Peng H.B. (2011). Axonal filopodial asymmetry induced by synaptic target. Mol. Biol. Cell.

[41] Li P.P., Zhou J.J., Meng M., Madhavan R., Peng H.B. (2012). Reciprocal regulation of axonal filopodia and outgrowth during neuromuscular junction development. PLoS ONE.

[42] Bertolesi G.E., Su H.Y., Michaiel G., Dueck S.M., Hehr C.L., McFarlane S. (2011). Two promoters with distinct activities in different tissues drive the expression of heparanase in Xenopus. Dev. Dyn..

[43] Bertolesi G.E., Michaiel G., McFarlane S. (2008). Two heparanase splicing variants with distinct properties are necessary in early Xenopus development. J. Biol. Chem..

[44] Bilican B., Fiore-Heriche C., Compston A., Allen N.D., Chandran S. (2008). Induction of Olig2 precursors by FGF involves BMP signalling blockade at the Smad level. PLoS ONE.

[45] Del Rio T., Nishitani A.M., Yu W.M., Goodrich L.V. (2013). *In vivo* analysis of *Lrig* genes reveals redundant and independent functions in the inner ear. PLoS Genet..

[46] Rondahl V., Holmlund C., Karlsson T., Wang B., Faraz M., Henriksson R., Hedman H. (2013). Lrig2-deficient mice are protected against PDGFB-Induced glioma. PLoS ONE.

[47] Hufton A.L., Vinayagam A., Suhai S., Baker J.C. (2006). Genomic analysis of Xenopus organizer function. BMC Dev. Biol..

[48] Fowler C.J., Griffiths D., de Groat W.C. (2008). The neural control of micturition. Nat. Rev. Neurosci..

[49] Weber S., Thiele H., Mir S., Toliat M.R., Sozeri B., Reutter H., Draaken M., Ludwig M., Altmüller J., Frommolt P. (2011). Muscarinic acetylcholine receptor M3 mutation causes urinary bladder disease and a prune -belly-like syndrome. Am. J. Hum. Genet.

[50] Cruz C.D., McMahon S.B., Cruz F. (2006). Spinal ERK activation contributes to the regulation of bladder function in spinal cord injured rats. Exp. Neurol..

[51] Jones E.A. (2005). Xenopus: a prince among models for kidney development. J. Am. Soc. Nephrol..

[52] Hilton E.N., Manson F.D., Urquhart J.E., Johnston J.J., Slavotinek A.M., Hedera P., Stattin E.L., Nordgren A., Biesecker L.G., Black G.C. (2007). Left-sided embryonic expression of the BCL-6 corepressor, BCOR, is required for vertebrate laterality determination. Hum. Mol. Genet..

[53] Nieuwkoop P.D., Faber J. (1967). Normal Table of Xenopus laevis (Daudin).

